# Epstein–Barr virus-driven immunosuppression in nasopharyngeal carcinoma: a comprehensive review of viral mechanisms, spatial tumor ecosystems, and precision therapeutics

**DOI:** 10.3389/fimmu.2026.1875687

**Published:** 2026-06-17

**Authors:** Huiwen Qu, Panpan Zhang, Yemei Tang, Tao Chang

**Affiliations:** Department of Otorhinolaryngology, Suzhou Ninth People’s Hospital, Suzhou, Jiangsu, China

**Keywords:** biomarkers, EBV lytic−phase variation, Epstein-Barr virus, immunotherapy, nasopharyngeal carcinoma, precision oncology, spatial omics, systematic review

## Abstract

**Background:**

EBV infection is the defining etiological factor in nasopharyngeal carcinoma (NPC), yet how viral factors systematically remodel the tumor immune microenvironment (TME) to sustain immunosuppression remains incompletely characterized. Existing reviews lack an integrated synthesis of viral mechanisms, TME spatial architecture, and therapeutic translation.

**Methods:**

We conducted a comprehensive literature search across PubMed, Embase, and Web of Science from inception to December 2025, with an update check to May 2026, following PRISMA guidelines. Given the broad scope, a narrative synthesis was adopted rather than a formal systematic review. Two reviewers independently screened 4,235 records, and 182 studies were included. Methodological quality was assessed using Cochrane RoB 2 and Newcastle−Ottawa tools, with detailed risk−of−bias summaries provided in the Supplementary Materials.

**Results:**

EBV establishes hierarchical immunosuppression in NPC. Latent proteins LMP1, LMP2A, and EBNA1, together with non−coding RNAs (BART miRNAs, EBERs), constitutively activate NF−κB, PI3K/AKT/mTOR, and JAK/STAT pathways; LMP1 further promotes exosomal secretion and metabolic reprogramming that expands myeloid−derived suppressor cells. Lytic−phase genetic polymorphisms in BALF2, BZLF1, and BRLF1 are associated with differential immune signatures, though these associations remain correlative and lack functional validation. Based on limited spatial profiling studies, the TME can be provisionally conceptualized as five distinct immunosuppressive niches—immune−excluded fibrotic stroma, immunosuppressive interface, tertiary lymphoid structures, vascular niches, and hypoxic tumor cores. Anti−PD−1−based chemo−immunotherapy achieves 20–91% objective response rates and is now the first−line standard for recurrent/metastatic disease, as established by the JUPITER−02 and RATIONALE−309 trials. EBV−directed adoptive cell therapies, therapeutic vaccines, lytic induction, and stromal modulators have shown early promise, although definitive efficacy data are still lacking. Biomarker integration—including plasma EBV DNA, viral genetic variants, spatial omics, and liquid biopsy—offers potential for patient stratification, yet most emerging markers remain investigational.

**Conclusions:**

This comprehensive review provides an evidence−based framework linking EBV latent and lytic mechanisms to TME remodeling and precision therapeutics. Key limitations include over−reliance on descriptive studies and insufficient functional validation of viral polymorphisms. Future research should prioritize spatial multi−omics, isogenic viral systems, humanized models, and adaptive trial designs to advance mechanism−driven therapy.

**Systematic review registration:**

https://www.crd.york.ac.uk/PROSPERO/, identifier CRD420261421334.

## 1 Introduction

The intricate interplay between specific viral infections and the development of human cancers offers a profound opportunity to dissect the fundamental processes of oncogenesis and immune evasion (1, 2). Among these, nasopharyngeal carcinoma (NPC), particularly the non-keratinizing subtype prevalent in regions such as Southern China and Southeast Asia, stands out due to its almost invariable association with Epstein-Barr virus (EBV) infection (3, 4). This robust etiological link positions NPC as an exceptional and compelling model for investigating how a virus can act not merely as an initiating factor, but as a continuous, master regulatory force that shapes the entire trajectory of a tumor (5, 6).

The pathogenesis of EBV-associated NPC is best understood as a chronic narrative of co-evolution and dynamic conflict. It represents a prolonged struggle between the virus’s sophisticated strategies for persistent latent infection and the host’s multilayered immune surveillance mechanisms (7–10). Recent years have witnessed a technological revolution in the tools available for such investigation. Innovations in single-cell multi-omics, spatial transcriptomics, and high-dimensional proteomics have begun to decode the staggering complexity of the NPC tumor ecosystem (11–14). These advanced methodologies reveal that EBV’s role extends far beyond the classical view of expressing a handful of oncogenic proteins. Instead, it involves a systematic and profound reprogramming of the host cell’s transcriptomic, epigenomic, and metabolic states (15). Furthermore, the virus orchestrates a deliberate, large-scale engineering of the surrounding tumor immune microenvironment (TME), effectively creating a sanctuary of immunosuppression that facilitates tumor survival and progression (16).

Despite the growing body of literature on EBV and NPC, significant gaps remain in our understanding of how viral factors specifically sculpt the immune landscape. While numerous narrative reviews have explored various facets of EBV biology (17–19), they often lack a focused, critical synthesis of the evidence linking viral mechanisms to TME remodeling and therapeutic vulnerabilities. Notably, emerging data on EBV strain/subtype variations—particularly polymorphisms in lytic−phase genes such as BALF2, BZLF1, and BRLF1—suggest that viral genetic diversity may influence immune signatures and clinical outcomes, yet this dimension has not been systematically integrated into existing frameworks (20, 21). Furthermore, many reviews have adopted a descriptive rather than critical stance, failing to evaluate the methodological quality of primary studies or to identify persistent knowledge gaps (22, 23). A comprehensive review is therefore essential to consolidate this rapidly expanding body of knowledge, distinguish robust findings from preliminary observations, critically appraise the evidence base, and identify clear pathways for future research and clinical translation (24, 25).

This comprehensive review aims to address this gap by achieving four primary objectives. First, to synthesize the existing evidence on how EBV latent proteins and non-coding RNAs hijack critical host cellular signaling and immune surveillance pathways, with particular emphasis on mechanisms directly relevant to TME remodeling (26, 27). Second, to characterize the contribution of EBV lytic−phase genetic variation—including but not limited to BALF2, BZLF1, and BRLF1 polymorphisms—to immune modulation and clinical outcomes, moving beyond a narrow focus on a single gene (28–30). Third, to evaluate the preclinical rationale and clinical efficacy of emerging therapeutic strategies that target these viral dependencies and immune resistance mechanisms, including a critical assessment of trial quality and generalizability (31, 32). Fourth, to summarize the current landscape and future potential of biomarkers, from established tools like plasma EBV DNA to cutting-edge spatial omics and liquid biopsy, for advancing precision oncology in NPC, with attention to emerging evidence on viral genetic determinants (33–36). By bridging fundamental virology with translational and clinical advances through a comprehensive and critical lens, this review seeks to outline a coherent and evidence-based roadmap for developing more effective, precise interventions against EBV-driven NPC.

## 2 Literature search and selection strategy

This comprehensive review was conducted based on a systematic literature search adhering to the principles of the Preferred Reporting Items for Systematic Reviews and Meta-Analyses (PRISMA) guidelines. However, given the breadth of the topic spanning molecular mechanisms, tumor microenvironment characterization, therapeutic strategies, and biomarker development, a narrative synthesis approach was adopted. The review protocol was not prospectively registered.

### 2.1 Search scope and eligibility criteria

To ensure a focused yet comprehensive synthesis, the following eligibility criteria were applied.

#### 2.1.1 Inclusion criteria

Studies were included if they met the following criteria: (1) Original research articles encompassing basic science (*in vitro*, *in vivo*), translational, or clinical studies (phase I-III trials, cohort studies); (2) Explicit investigation of the role of EBV in the context of NPC pathogenesis, immune microenvironment, therapeutic response, or biomarker development; with priority given to studies examining immunomodulatory mechanisms; (3) Publication in the English language; (4) Availability of full text.

#### 2.1.2 Exclusion criteria

Studies were excluded based on the following: (1) Focus on other EBV-associated malignancies (e.g., lymphoma, gastric carcinoma) without specific NPC data; (2) Conference abstracts, editorials, commentaries, or case reports with fewer than five patients, unless they presented unique and foundational mechanistic insights not available elsewhere; (3) Studies with insufficient methodological detail to assess quality or validity; (4) Duplicate publications reporting on the same cohort without novel analysis.

### 2.2 Information sources and search strategy

A comprehensive search was performed to minimize the risk of missing relevant evidence. The electronic bibliographic databases PubMed/MEDLINE, Embase, and Web of Science Core Collection were searched from their inception to December 31, 2025. The primary search was conducted up to this date; a subsequent rapid literature checked up to May 2026 did not reveal any new practice-changing clinical trial results that would alter the main conclusions of this review.

No start date restriction was applied to capture foundational studies. The search strategy was developed iteratively by the review team with input from a medical librarian to ensure sensitivity and specificity. It combined controlled vocabulary (MeSH terms in PubMed, Emtree in Embase) and free-text keywords related to the core concepts: “Epstein-Barr virus”, “nasopharyngeal carcinoma”, “tumor microenvironment”, “immunotherapy”, “viral strain”, “BALF2”, “genetic variation”, and key pathways (e.g., “NF-kappa B”, “PI3K”, “PD-L1”). Boolean operators (AND, OR) were used to link concepts. The full, detailed search strategy for PubMed is provided in **Supplementary Table 1**. To complement the database search, the reference lists of all included studies and relevant review articles were manually scanned for additional eligible publications (snowballing).

### 2.3 Study selection process

The study selection process was carried out independently by two reviewers (H.Q. and P.Z.) to minimize bias and error. All records identified through the database searches were imported into the Covidence systematic review software for deduplication and management. The selection process occurred in two sequential stages:

Title and Abstract Screening: The two reviewers independently screened the titles and abstracts of all unique records against the predefined eligibility criteria. Records that clearly did not meet the criteria were excluded. Those deemed potentially relevant or where relevance was uncertain proceeded to full-text review.

**Figure 1:**
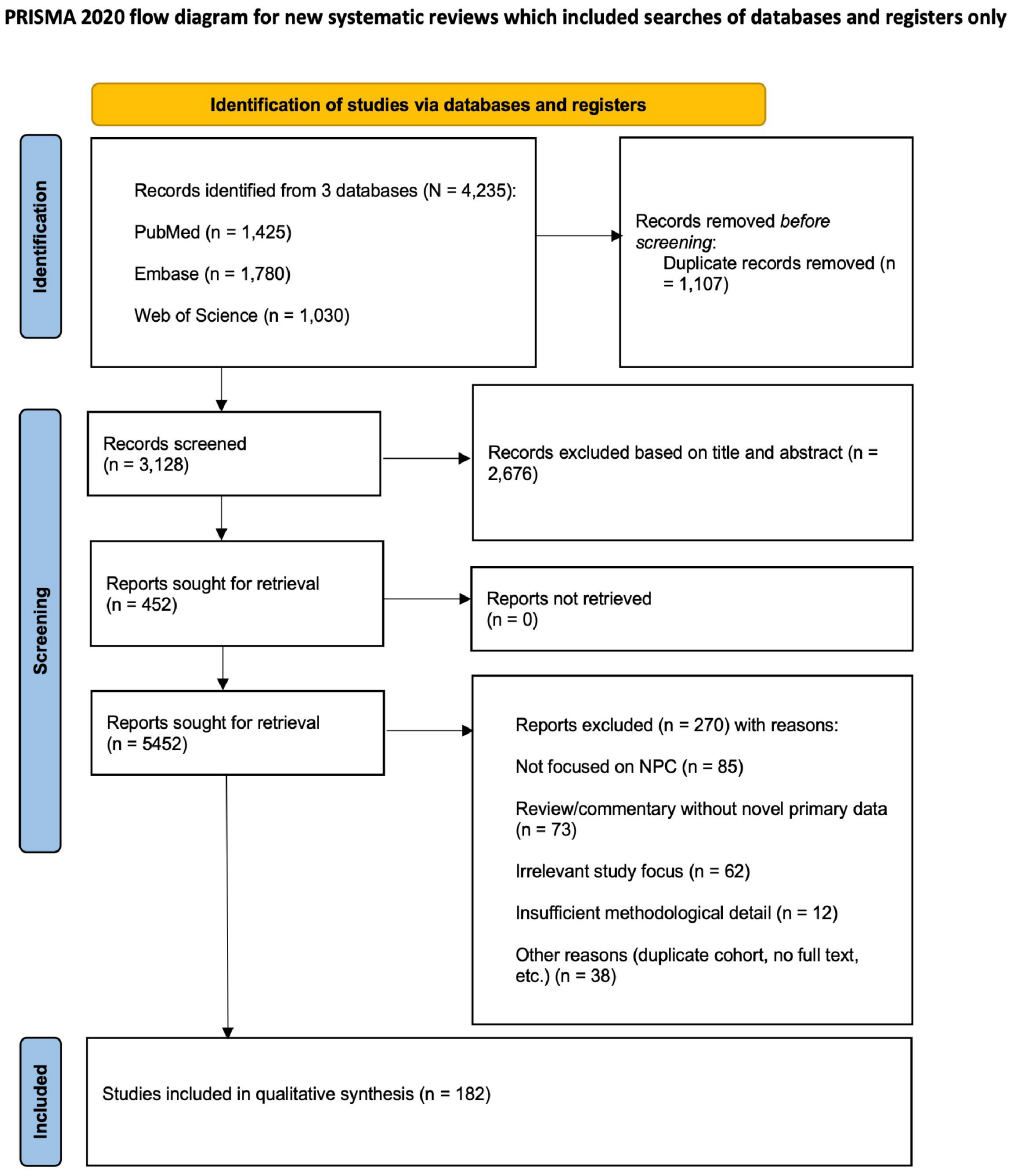
Literature search and selection process based on PRISMA guidelines. The flow diagram details the identification, screening, eligibility assessment, and inclusion stages of the comprehensive review. A total of 4,235 records were identified from three electronic databases. After removing 1,107 duplicates, 3,128 unique records underwent title and abstract screening, of which 2,676 were excluded as clearly irrelevant. Of the 452 full-text articles assessed for eligibility, 270 were excluded for the reasons specified, and a further 12 studies were excluded due to insufficient methodological detail. Ultimately, 182 studies were included in the qualitative synthesis. EBV, Epstein–Barr virus; NPC, nasopharyngeal carcinoma.

Full-Text Review: The full-text articles of all records passing the initial screen were retrieved and independently assessed by the same two reviewers for final eligibility. At both stages, any disagreements between the reviewers regarding the inclusion or exclusion of a study were resolved through discussion. If consensus could not be reached, a third senior reviewer (T.C.) was consulted to make the final decision. The reasons for excluding studies at the full-text stage were documented. The entire selection process is summarized in a flow diagram (**Figure 1**), which was constructed based on PRISMA guidelines to ensure transparency.

### 2.4 Data extraction and management

Data from each included study were extracted independently by the two reviewers using a standardized, piloted data extraction form created in Microsoft Excel. The form captured the following key information:

Study Characteristics: First author, publication year, country of origin, study design (e.g., *in vitro* experiment, animal study, retrospective cohort, clinical trial), and sample size.

Participant/Cell Model Details: For clinical/preclinical studies, relevant characteristics (e.g., cell line used, animal model, patient disease stage, EBV status, viral strain information if available).

EBV Factors Investigated: Specific viral latent proteins, non-coding RNAs, viral life cycle phases, or strain/subtype characteristics studied.

Key Findings: Detailed results related to molecular mechanisms (with emphasis on immunomodulation), TME characterization, therapeutic efficacy/mechanism, or biomarker performance, including quantitative data (e.g., hazard ratios, response rates, p-values) where available.

Conclusions: Authors’ main conclusions relevant to the review objectives.

After independent extraction, the two reviewers compared their completed forms. Any discrepancies in the extracted data were identified and resolved by referring back to the original publication and through discussion, ensuring accuracy and consistency.

### 2.5 Quality appraisal and risk of bias assessment

Although this is a comprehensive review with narrative synthesis, the methodological quality of included studies was assessed to inform the critical appraisal of the evidence. For preclinical laboratory studies (*in vitro* and *in vivo*), a customized checklist based on established principles was used, evaluating elements such as blinding during outcome assessment, whether experiments were replicated, the appropriateness of controls (e.g., isogenic EBV-negative controls, viral strain-matched comparisons), and the characterization of cell lines/models (7, 8). For clinical trials, the revised Cochrane Risk of Bias tool (RoB 2) was used for randomized trials, assessing bias arising from the randomization process, deviations from intended interventions, missing outcome data, outcome measurement, and selection of the reported result. For observational clinical studies (e.g., cohort studies), the Newcastle-Ottawa Scale was employed to evaluate selection of study groups, comparability of groups, and ascertainment of exposure/outcome. A critical component of our assessment was the evaluation of whether studies provided sufficient methodological detail to support causal inferences about EBV’s role in immune modulation. The detailed risk-of-bias assessments are provided in **Supplementary Table 2**. The overall strength of the body of evidence for major conclusions was qualitatively graded, considering the consistency, precision, and directness of the findings across studies. It is important to note that most conclusions in this review are drawn from heterogeneous observational and early-phase studies, and the overall evidence level should be considered moderate to low unless otherwise specified.

### 2.6 Data synthesis

**Figure 2:**
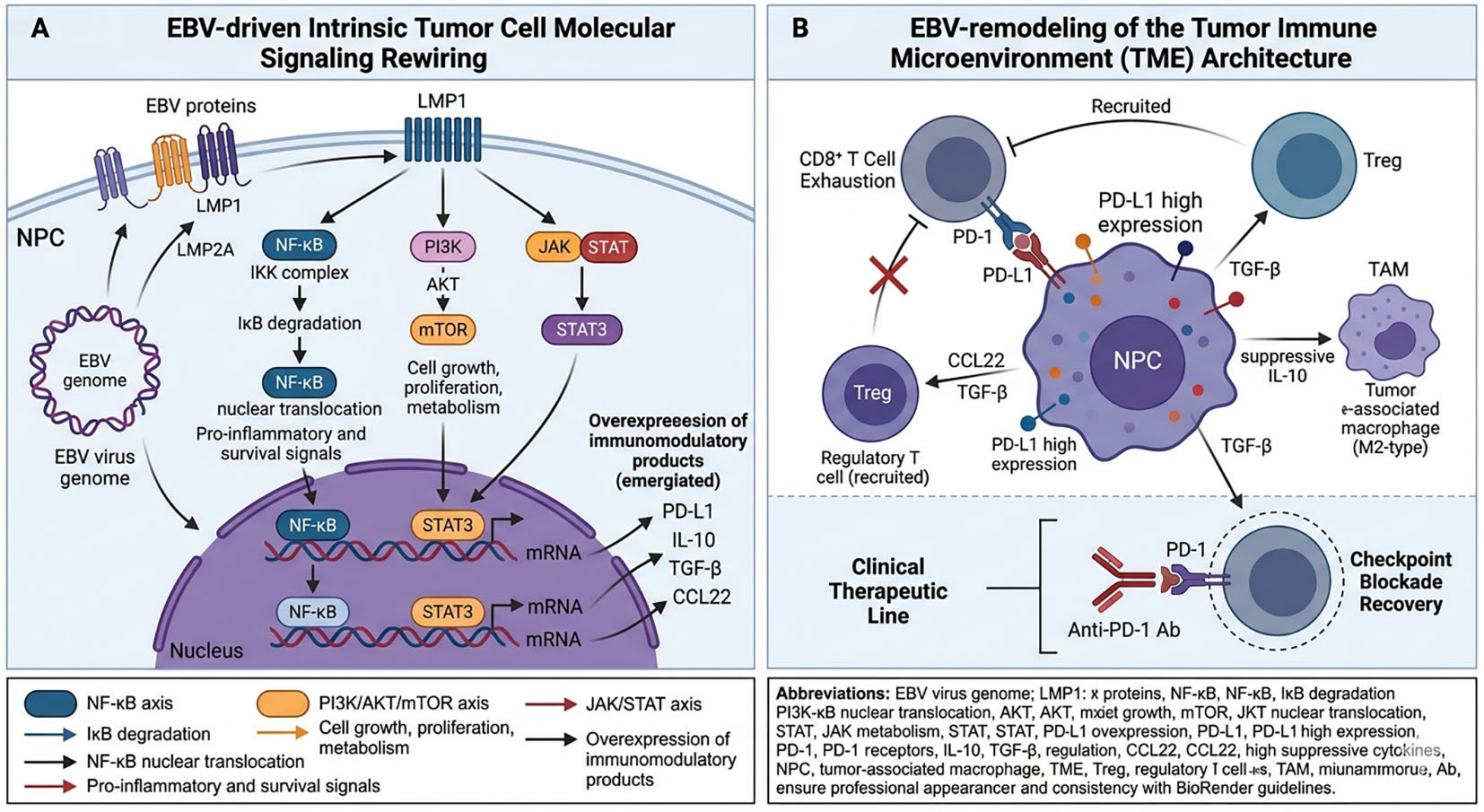
EBV-driven signaling rewiring and immune microenvironment remodeling in NPC. **(A)** In nasopharyngeal carcinoma (NPC) cells, latent EBV infection drives tumor-intrinsic signaling rewiring mainly through the viral oncoproteins LMP1 and LMP2A. LMP1 constitutively activates the NF-κB pathway, promoting pro-survival and pro-inflammatory signaling, whereas LMP2A activates the PI3K/AKT/mTOR and JAK/STAT3 pathways to support proliferation, metabolic reprogramming, and immune evasion. These convergent signals induce the expression of immunosuppressive mediators, including PD-L1, IL-10, TGF-β, and CCL22. **(B)** Beyond tumor cells, EBV-positive NPC remodels the tumor microenvironment into an immunosuppressive niche. PD-L1 expression on NPC cells promotes CD8^+^ T-cell exhaustion through PD-1/PD-L1 signaling; CCL22 recruits Treg cells, which further suppress effector T-cell activity through TGF-β; and IL-10 contributes to polarization of M2-like tumor-associated macrophages (TAMs). Together, these interactions establish a functionally suppressed microenvironment, while anti-PD-1/PD-L1 therapy may help restore antitumor immunity. Abbreviations: EBV, Epstein-Barr virus; NPC, Nasopharyngeal carcinoma; LMP1/2A, Latent membrane protein 1/2A; IKK, IκB kinase; NF-κB, Nuclear factor kappa-light-chain-enhancer of activated B cells; PI3K, Phosphoinositide 3-kinase; mTOR, Mammalian target of rapamycin; STAT3, Signal transducer and activator of transcription 3; PD-L1, Programmed death-ligand 1; PD-1, Programmed cell death protein 1; Treg, Regulatory T cell; TAM, Tumor-associated macrophage; CCL22, C-C motif chemokine ligand 22; TGF-β, Transforming growth factor beta; IL-10, Interleukin-10.

Given the anticipated and observed significant heterogeneity across the included studies in terms of designs, experimental models, interventions, and outcome measures, a quantitative meta-analysis was not performed. Instead, a qualitative narrative synthesis was conducted. Therefore, a qualitative narrative synthesis was performed. The synthesis was structured thematically around the primary review objectives. Findings from the included studies were grouped into logical categories: viral mechanisms (with a dedicated subsection on strain variation), TME composition and spatial architecture, therapeutic strategies, and biomarkers. Within each category, evidence was summarized, similarities and differences between studies were highlighted, methodological limitations were critically appraised, and the results were presented in a logical narrative flow. Throughout the synthesis, particular attention was paid to distinguishing between well-validated findings and preliminary or hypothesis-generating observations, and to appropriately qualifying the strength of evidence for each major conclusion. To aid clarity and provide concise overviews, evidence was also synthesized and presented in tabular form, resulting in four summary tables: **Table 1** (EBV viral factors—expanded to include strain variation), **Table 2** (Immune cell subsets in TME), **Table 3** (Clinical outcomes of ICIs—updated to include tislelizumab trials), and **Table 4** (Biomarkers—expanded to include BALF2). To enhance conceptual clarity and provide a visual synthesis of the complex mechanistic and spatial relationships discussed in Sections 3.2 and 3.3, a two-panel mechanistic diagram (**Figure 2**) was created. Panel A illustrates the hierarchical EBV signaling cascade from latent proteins through downstream pathways to functional outputs, while Panel B depicts a proposed model of five recurrent spatial cellular neighborhoods within the NPC tumor microenvironment and their interrelationships.

**Table 1:** EBV latent and lytic phase viral factors and their major functions in NPC.

Viral factor	Molecular features	Targeted pathways	Immunomodulatory functions	Prognostic associations	Strain variant implications	References
EBNA1	DNA-binding protein with Gly-Ala repeat domain	Viral episome maintenance, chromosomal binding	Inhibits antigen processing and presentation via its Gly-Ala repeat domain, thereby reducing CD8^+^ T cell recognition	Associated with viral persistence and genomic instability	Sequence polymorphisms may affect protein stability and immune recognition	(7, 18, 33, 35)
LMP1	Constitutively active TNF receptor homolog (CTAR1/CTAR2 domains)	NF-κB (canonical/non canonical), JAK/STAT, PI3K/AKT	Upregulates PD-L1 and secretes pro-inflammatory cytokines (IL-6, IL-8); promotes Treg and MDSC expansion via a glycolytic switch; is secreted in exosomes to exert paracrine immunosuppressive effects	High expression correlates with advanced stage and poor survival	Variants with differential signaling potency may alter immune microenvironment	(19, 20, 27, 30, 31)
LMP2A	ITAM containing B cell receptor mimic	PI3K/AKT/mTOR, MAPK	Drives metabolic reprogramming (Warburg effect), inhibits autophagy, and promotes vasculogenic mimicry through VEGFA/VEGFR1 and ERK pathways, contributing to immune escape	Associated with metastasis and therapy resistance	Polymorphisms in ITAM motifs may affect signaling intensity	(36–40)
BART miRNAs	>40 viral miRNAs from BamHI A region	Post transcriptional gene silencing	Target PUMA, p53, ICAM-1, MICB, and TAP2 to suppress apoptosis, antigen presentation, and NK cell recognition; miR-BART1-5p regulates vasculogenic mimicry via Spry2	Specific miRNAs correlate with immune evasion and poor response to immunotherapy	miRNA expression levels may vary by strain, affecting immune modulation	(40–45)
EBERs	Non coding, non polyadenylated RNAs (most abundant transcripts)	RIG I/NF κB	Induce low-level type I interferon and NF-κB signaling via RIG-I, paradoxically promoting cell survival under stress and contributing to a chronic inflammatory milieu	Ubiquitous in latency; clinical significance as diagnostic marker	Sequence conservation suggests limited strain variation	(46–49)
BZLF1 (lytic)	Immediate early transcription factor (Zta)	Lytic cycle reactivation	Induces expression of immunogenic lytic antigens; naturally occurring variants may affect reactivation efficiency, but the functional consequences on immune phenotypes remain unproven	Specific variants enriched in endemic NPC; linked to elevated reactivation	Polymorphisms associated with differential transactivation capacity and reactivation potential	(50–53)
BRLF1 (lytic)	Immediate early transcription factor (Rta)	Lytic cycle reactivation	Co-activates lytic gene expression; promoter polymorphisms are associated with altered transcriptional activity in vitro, though in vivo immune relevance is not established	Variants may affect response to lytic induction therapy	Sequence variations influence transcriptional activity	(50, 51)
BALF2 (lytic)	Single stranded DNA binding protein	Viral DNA replication	Emerging evidence links certain polymorphisms to differential immune-related gene expression and interferon signaling; however, these associations are purely correlative and require functional validation before any clinical application	Specific variants associated with altered transcriptomic profiles and clinical outcomes	Polymorphisms correlate with distinct immune-related gene expression patterns; high-risk strains alter interferon signaling	(1, 20, 54, 55)
EBNA1	DNA-binding protein with Gly-Ala repeat domain	Viral episome maintenance, chromosomal binding	Inhibits antigen processing and presentation via its Gly-Ala repeat domain, thereby reducing CD8^+^ T cell recognition	Associated with viral persistence and genomic instability	Sequence polymorphisms may affect protein stability and immune recognition	(7, 18, 33, 35)
LMP1	Constitutively active TNF receptor homolog (CTAR1/CTAR2 domains)	NF-κB (canonical/non canonical), JAK/STAT, PI3K/AKT	Upregulates PD-L1 and secretes pro-inflammatory cytokines (IL-6, IL-8); promotes Treg and MDSC expansion via a glycolytic switch; is secreted in exosomes to exert paracrine immunosuppressive effects	High expression correlates with advanced stage and poor survival	Variants with differential signaling potency may alter immune microenvironment	(19, 20, 27, 30, 31)
LMP2A	ITAM containing B cell receptor mimic	PI3K/AKT/mTOR, MAPK	Drives metabolic reprogramming (Warburg effect), inhibits autophagy, and promotes vasculogenic mimicry through VEGFA/VEGFR1 and ERK pathways, contributing to immune escape	Associated with metastasis and therapy resistance	Polymorphisms in ITAM motifs may affect signaling intensity	(36–40)
BART miRNAs	>40 viral miRNAs from BamHI A region	Post transcriptional gene silencing	Target PUMA, p53, ICAM-1, MICB, and TAP2 to suppress apoptosis, antigen presentation, and NK cell recognition; miR-BART1-5p regulates vasculogenic mimicry via Spry2	Specific miRNAs correlate with immune evasion and poor response to immunotherapy	miRNA expression levels may vary by strain, affecting immune modulation	(40–45)
EBERs	Non coding, non polyadenylated RNAs (most abundant transcripts)	RIG I/NF κB	Induce low-level type I interferon and NF-κB signaling via RIG-I, paradoxically promoting cell survival under stress and contributing to a chronic inflammatory milieu	Ubiquitous in latency; clinical significance as diagnostic marker	Sequence conservation suggests limited strain variation	(46–49)
BZLF1 (lytic)	Immediate early transcription factor (Zta)	Lytic cycle reactivation	Induces expression of immunogenic lytic antigens; naturally occurring variants may affect reactivation efficiency, but the functional consequences on immune phenotypes remain unproven	Specific variants enriched in endemic NPC; linked to elevated reactivation	Polymorphisms associated with differential transactivation capacity and reactivation potential	(50–53)
BRLF1 (lytic)	Immediate early transcription factor (Rta)	Lytic cycle reactivation	Co-activates lytic gene expression; promoter polymorphisms are associated with altered transcriptional activity in vitro, though in vivo immune relevance is not established	Variants may affect response to lytic induction therapy	Sequence variations influence transcriptional activity	(50, 51)
BALF2 (lytic)	Single stranded DNA binding protein	Viral DNA replication	Emerging evidence links certain polymorphisms to differential immune-related gene expression and interferon signaling; however, these associations are purely correlative and require functional validation before any clinical application	Specific variants associated with altered transcriptomic profiles and clinical outcomes	Polymorphisms correlate with distinct immune-related gene expression patterns; high-risk strains alter interferon signaling	(1, 20, 54, 55)

**Table 2:** Immune cell subsets in the NPC tumor microenvironment and their functional states.

Cell type	Phenotypic markers	Functional state in NPC TME	Immunosuppressive mechanisms	Spatial localization (neighborhood)	Therapeutic targeting strategies	References
CD8^+^ T cells	PD-1^+^, TIM-3^+^, LAG-3^+^, TIGIT^+^	Terminally exhausted (TOX^+^, NR4A^+^); progenitor exhausted subset	Loss of effector cytokines, metabolic defects, arrested proliferation	Trapped in Immune-Excluded Fibrotic Stromal Neighborhood (excluded) or engaged at Interface (exhausted)	PD-1/PD-L1 blockade, combination with anti-TIM-3/LAG-3	(56–62)
Regulatory T cells	CD4^+^CD25^+^FOXP3^+^	Highly suppressive, correlated with plasma EBV DNA load	IL-2 consumption, granzyme/perforin-mediated killing, IL-10/TGF-β secretion ; CD70-CD27 co-stimulation may further promote Treg expansion and suppressive function	Enriched at Vascular Niche and Interface	Anti-CTLA-4, CCR4 antagonists, low-dose cyclophosphamide , CD70/CD27 blockade	(50, 63–71)
Tumor associated macrophages	CD68^+^CD163^+^, SPP1^+^, TREM2^+^, C1Q^+^	M2-like polarization, M1-M2 coupled pattern	ARG1-mediated L-arginine depletion, PD-L1/VISTA expression, IL-10/TGF-β secretion	Interface and Immune-Excluded Fibrotic Stromal Neighborhood	CSF-1R inhibitors, CD47 blockade, repolarization agents	(31, 40, 69, 72–75)
Myeloid derived suppressor cells	CD33^+^CD11b^+^HLA-DR^-^	Expanded via LMP1–glycolysis axis	Inhibition of T cell proliferation, promotion of Treg differentiation	Vascular Niche and Interface	COX-2 inhibitors, PDE5 inhibitors, arginase inhibitors	(31, 40, 76–78)
Cancer associated fibroblasts	α-SMA^+^, FAP^+^	Activated by TGF-β/PDGF; form stromal barrier	ECM deposition (collagen, fibronectin), physical exclusion of T cells, CXCL12/VEGF secretion	Immune-Excluded Fibrotic Stromal Neighborhood (dominant)	FAP-targeted therapies, TGF-β inhibitors, LOXL2 inhibitors	(70, 79–83)
B cells	CD19^+^, CD20^+^	Heterogeneous; IgD^-^CD27^-^ double-negative subset correlates with poor outcome	IL-10 production via EBV-induced miR-21, suppression of CD8^+^ T cells	TLS (organized) or scattered (Interface)	Anti-CD20 (rituximab), BTK inhibitors, BAFF/APRIL blockade	(84–89)
Natural killer cells	CD56^+^, NKG2D^+^	Exhausted phenotype (TIGIT^+^, LAG3^+^)	Impaired cytotoxicity, reduced IFN-γ production	Scattered, often Vascular Niche	Anti-TIGIT, IL-15 superagonists, CAR-NK cell therapy	(61, 62, 90–94)

**Table 3:** Key clinical outcomes of immune checkpoint inhibitors in recurrent/metastatic NPC.

Trial/regimen	Phase	Patient population	ORR	Median PFS	Median OS	Key findings	Critical appraisal	References
KEYNOTE-028 (Pembrolizumab)	I	PD-L1^+^ R/M NPC (n=27)	26%	6.5 months	16.5 months	First demonstration of anti-PD-1 activity in NPC	Small sample, single arm, selected for PD-L1^+^	(8)
NCT02339558 (Nivolumab)	II	R/M NPC (n=44)	20.5%	5.6 months	17.1 months	Durable responses in heavily pretreated patients	Single arm, heterogeneous prior treatments	(9)
CAPTAIN-1st (Camrelizumab + Chemo)	III	Untreated R/M NPC (n=134)	91%	10.4 months	Not reached	Established chemo-immunotherapy as new standard	Randomized, open-label; primary endpoint met	(10)
JUPITER-02 (Toripalimab + Chemo)	III	Untreated R/M NPC (n=146)	77.4%	11.7 vs 8.0 months (HR=0.52)	Not reached (HR=0.60)	Significant PFS benefit, approved as first-line therapy	Randomized, double-blind, robust design	(31)
RATIONALE-309 (Tislelizumab + Chemo)	III	Untreated R/M NPC (n=263)	69.5%	9.6 vs 7.4 months (HR=0.50)	Not reached	Added tislelizumab to effective ICI options; cost-effective	Large randomized trial, confirms class effect	(32, 95)
Camrelizumab + Apatinib	II	R/M NPC (n=23)	65.2%	10.4 months	Not reached	Synergy between ICI and anti-angiogenic agent	Small single arm, hypothesis-generating only	(96)

All ORR and survival data are from published reports; some confidence intervals and final OS data remain pending. The RATIONALE-309 ORR was updated to 69.5% based on the latest follow-up.

**Table 4:** EBV-related biomarkers and their clinical significance in NPC.

Biomarker category	Specific marker	Detection method	Clinical utility	Predictive/prognostic value	Evidence level	References
Virological	Plasma EBV DNA	qPCR, ddPCR	Diagnosis, staging, monitoring response, surveillance	High baseline load = poor prognosis; clearance during therapy predicts better outcome	High (prospectively validated)	(2, 68, 97)
	EBV fragmentomics	Next generation sequencing	Distinguish apoptosis vs necrosis, monitor therapy-induced cell death	Specific fragmentation patterns may predict response to immunotherapy	Moderate (exploratory)	(1, 98)
	EBV lytic-phase variants (BALF2, BZLF1, BRLF1)	Sequencing	Potential for patient stratification based on immune signature and reactivation potential	Specific variants associated with differential immune gene expression, altered interferon signaling, and elevated reactivation; all associations are correlative, and no functional validation exists	Low (early discovery) ; correlative only, no functional validation	(1, 20, 50, 54, 55)
Serological	VCA IgA, EBNA1 IgA, EA IgA	ELISA, immunofluorescence	Screening, early diagnosis	Combination improves sensitivity/specificity	High (screening)	(99–102)
	Anti-BNLF2b total antibody (P85-Ab)	ELISA	Early detection in screening programs	Higher sensitivity (97.9%) and specificity (98.3%) than traditional antibodies	High (large validation study)	(103–106)
Tissue-based	PD-L1 CPS	IHC (22C3, SP263)	Patient selection for anti-PD-1 therapy	CPS ≥1 required for pembrolizumab; higher CPS may correlate with better response	Moderate (companion diagnostic but imperfect)	(31, 107)
	Tertiary lymphoid structures (TLS)	Multiplex IHC (CD20, CD21, CXCL13)	Prognosis and response prediction	Presence correlates with improved survival and better response to ICI; maturation status (germinal center presence) likely adds predictive value	Moderate (retrospective cohorts)	(4, 6, 57, 62, 108–110)
	Tumor-infiltrating Tregs (CD4^+^CD25^+^FOXP3^+^)	Multiplex IHC	Prognosis and immune monitoring	High density correlates with higher EBV DNA load and worse PFS	Moderate (correlative studies)	(63–66)
Liquid biopsy (emerging)	BRRF2^+^ extracellular vesicles	Immuno-qPCR	Predict response to anti-PD-1 therapy	High pre-treatment levels associated with poor response (stable/progressive disease)	Low (small studies)	(111, 112)
	Circulating tumor DNA (ctDNA)	NGS panel	Monitor clonal evolution, detect resistance mutations	Emerging mutations in JAK/STAT pathway may indicate acquired resistance	Moderate (proof of concept)	(1, 113, 114)
METABOLIC imaging	^68^Ga-DOTA-SSTR2 PET-CT	PET-CT imaging	Diagnosis, staging, follow-up	SSTR2 expression correlates with EBV positivity and tumor aggressiveness	Moderate (imaging biomarker)	(115–119)

## 3 Results

### 3.1 Study selection

The results of the comprehensive literature search and selection process are detailed in the flow diagram (**Figure 1**). The initial electronic database searches of PubMed, Embase, and Web of Science yielded a total of 4,235 records. After the removal of 1,107 duplicates, 3,128 unique records remained for title and abstract screening. Based on this screening, 2,676 records were excluded as clearly irrelevant. The remaining 452 full-text articles were retrieved and assessed in detail for eligibility. Of these, 270 were excluded with reasons, most commonly for not focusing on NPC (n=85), being a review/commentary without novel primary data (n=73), or having an irrelevant study focus (n=62). A further 12 studies were excluded due to insufficient methodological detail to assess quality, particularly regarding viral characterization or immune phenotyping. Ultimately, 182 studies satisfied all eligibility criteria and were included in the qualitative synthesis of this comprehensive review. The included studies encompassed a wide spectrum, from foundational mechanistic laboratory research to late-phase clinical trials. As detailed in **Supplementary Table 2**, the overall risk of bias in the included evidence ranges from low for pivotal randomized trials to high for many preclinical studies, and this variability was taken into account during the narrative synthesis.

### 3.2 Foundational mechanisms: viral hijacking of cellular machinery and immune surveillance

The synthesis of 65 studies focused on molecular pathogenesis provides robust evidence that EBV establishes a specific latent infection program (predominantly Latency II) within nasopharyngeal epithelial cells, characterized by the expression of a strategic repertoire of viral products: Epstein-Barr nuclear antigen 1 (EBNA1), the latent membrane proteins 1 and 2 (LMP1, LMP2A/B), the non-coding EBER RNAs, and the BamHI-A rightward transcript (BART) microRNAs (7–12). The coordinated action of these elements initiates and sustains a multi-decade campaign of cellular reprogramming. To enhance conceptual clarity, we present these mechanisms within a hierarchical framework (**Figure 2A**), distinguishing between primary viral effectors, downstream signaling cascades, and functional outputs relevant to immune modulation.

The latent membrane protein 1 (LMP1) is widely established as the principal viral oncoprotein and the master immunomodulator in NPC (18). Functioning as a constitutively active homolog of the CD40 receptor, LMP1’s cytoplasmic tail domains (CTAR1 and CTAR2) serve as platforms to recruit host tumor necrosis factor receptor-associated factors (TRAFs), leading to the perpetual activation of both the canonical and non-canonical NF-κB signaling pathways (19–22). This relentless NF-κB signaling drives a profound transcriptional reprogramming. It upregulates genes critical for cell survival (e.g., BCL-2), proliferation (e.g., cyclin D1), and invasion, while also stimulating the secretion of a broad spectrum of pro-inflammatory cytokines and chemokines such as IL-6, IL-8, and CCL20 (23–27). Furthermore, LMP1 is a potent activator of the JAK/STAT and PI3K/AKT/mTOR pathways, further entrenching proliferative and anti-apoptotic signals within the infected cell (28, 29). Critically, recent high-impact studies have expanded our understanding of LMP1’s role beyond cell-autonomous signaling to include paracrine immunomodulation. It has been shown that LMP1 can be secreted within exosomes and can physically interact with the glucose transporter GLUT1, stabilizing it and driving a glycolytic switch in tumor cells (30–32). This metabolic reprogramming, in turn, activates signaling nodes like the NLRP3 inflammasome, leading to the secretion of cytokines such as IL-1β and GM-CSF that promote the differentiation and expansion of immunosuppressive myeloid-derived suppressor cells (MDSCs) in the TME (31). This LMP1-glycolysis-MDSC axis represents a direct mechanistic link between viral oncoprotein expression and the construction of an immunosuppressive microenvironment.

Acting in concert with LMP1, latent membrane protein 2A (LMP2A) provides surrogate, tonic B-cell receptor-like signals through its immunoreceptor tyrosine-based activation motifs (ITAMs) (35). Recruitment of SYK and SRC family kinases by LMP2A leads to potent activation of the PI3K/AKT/mTOR axis, a central regulator of cell growth, metabolism, and survival (36, 37). This activation drives a profound metabolic reprogramming toward aerobic glycolysis (the Warburg effect), fueling the biosynthetic demands of rapid proliferation while creating a nutrient-depleted and acidic microenvironment hostile to immune effector cells (38). LMP2A has also been implicated in inhibiting autophagy, a process crucial for cellular quality control, potentially contributing to genomic instability (120, 121). Its spliced variant, LMP2B, is thought to fine-tune these oncogenic signals (39).

Beyond proteins, EBV deploys a sophisticated arsenal of non-coding RNAs. The EBV-encoded small RNAs (EBERs), the most abundant viral transcripts during latency, are non-translated but biologically active. They can bind to and activate the cytoplasmic RNA sensor RIG-I, leading to a low-level induction of type I interferon and NF-κB, which paradoxically may promote cell survival under stress conditions (46–49). The BART microRNAs represent a more nuanced layer of regulation. Over 40 viral miRNAs are processed from the BART transcripts and function as key agents of immune evasion and oncogenic tuning. For instance, miR-BART5-5p targets the pro-apoptotic protein PUMA, while miR-BART22 can suppress the stress ligand MICB, impairing natural killer (NK) cell recognition (41–45). This multi-pronged, miRNA-mediated strategy effectively cloaks the infected cell from both adaptive (CD8+ T cell) and innate (NK cell) immune surveillance.

A critical dimension of EBV’s strategy is its deep interplay with the host epigenetic machinery. The virus rewrites the epigenetic code to lock in its latent program. EBNA1 can recruit host DNA methyltransferases like DNMT3B to specific promoters, leading to CpG methylation and silencing of both viral lytic genes (e.g., BZLF1) and host tumor suppressor genes such as RASSF1A and p16INK4a (33, 34, 97, 122–125). Simultaneously, viral proteins like LMP1 can induce expression of the histone methyltransferase EZH2, which deposits repressive H3K27me3 marks on target genes (126, 127). This creates a state of “oncogenic addiction” and a persistent “viral epigenetic scar” (128–130). Recent studies have unveiled additional sophisticated mechanisms, such as the secretion of the EBV tegument protein BRRF2 within extracellular vesicles. When captured by immune cells like macrophages, BRRF2 inhibits the cGAS-STING innate immune sensing pathway by disrupting cGAS phase separation, representing a novel paracrine strategy for systemic immune evasion (111, 131–133).

Beyond the well−characterized latent gene products, an emerging area of investigation concerns the role of EBV lytic−phase genetic variation in modulating immune responses. Several studies have identified polymorphisms in viral lytic genes that may influence pathogenesis (20, 21, 50). Notably, variants in the BALF2 gene—which encodes a single-stranded DNA-binding protein essential for viral DNA replication—have been associated with differential immune signatures and clinical outcomes (54, 55). In a large−scale genomic analysis, Ding et al. demonstrated that specific BALF2 variants correlate with distinct transcriptomic profiles in NPC, including differential expression of immune−related genes and pathways (1). High−risk EBV strains, characterized by specific BALF2 polymorphisms, significantly alter NPC gene expression profiles, with particular impact on immune−related pathways and interferon signaling (54). Population−specific EBV sequence variations among Southeast Asian NPC patients reveal distinct viral evolutionary patterns and BALF2 diversity that may contribute to regional differences in tumor immunogenicity (55).

However, focusing solely on BALF2 would provide an incomplete picture of EBV’s lytic−phase diversity. Other lytic genes also exhibit clinically relevant polymorphisms. The BZLF1 gene, encoding the immediate−early protein Zta that initiates the lytic cascade, carries naturally occurring variants with differential transactivation capacity; certain BZLF1 variants are associated with elevated viral reactivation potential and are enriched in NPC−endemic regions (50). Similarly, BRLF1 polymorphisms have been linked to altered promoter activity and may influence the efficiency of lytic induction, with potential implications for lytic−induction therapy (50). These observations collectively underscore that EBV’s influence on immune modulation is not restricted to its latent program but extends to genetic variation within lytic−phase regulators.

These findings suggest that viral genetic diversity may contribute to inter-individual variability in tumor immunogenicity and response to immunotherapy. However, it is critical to emphasize that all current evidence linking BALF2, BZLF1, and BRLF1 polymorphisms to altered immune signatures is purely correlative and derived from descriptive sequencing or genome-wide association studies. No functional studies using isogenic viral mutants have yet demonstrated that these specific single-nucleotide polymorphisms directly cause the observed immune phenotypes, rather than simply being linked to them. Therefore, these genetic associations must be strictly viewed as hypothesis-generating and are not yet suitable for any form of clinical stratification or decision-making. Future research should prioritize isogenic viral systems to dissect whether and how specific polymorphisms causally impact immune modulation.

In addition to viral genetic variation, other EBV−induced vulnerabilities have been characterized. Vasculogenic mimicry (VM)—the formation of fluid−conducting channels by tumor cells—has been proposed as a mechanism of therapy resistance. The initial report of EBV-driven VM via the PI3K/AKT/mTOR/HIF-1α axis was subsequently retracted due to image errors and cell line contamination concerns (134, 135). Consequently, the specific findings of that study are unreliable and must be entirely disregarded. Nevertheless, multiple independent investigations have provided evidence supporting a role for EBV in VM formation through alternative molecular mechanisms. Xu et al. demonstrated that LMP1 promotes VM via VEGFA/VEGFR1 signaling (8). Wang et al. showed that EBV−miR−BART1−5p contributes to VM through Spry2−dependent regulation of the PI3K/AKT/mTOR/HIF1−α pathway, and that exosome−delivered antagomiRs against miR−BART1−5p inhibit both VM and angiogenesis in NPC models (40). Additionally, Xiang et al. (2024) established that EBV−associated epithelial cancers promote VM formation through secretory cross−talk with the immune microenvironment, providing a mechanistic link between viral infection, VM, and immunosuppression (136). Collectively, these independent studies suggest that the broader concept of EBV-associated VM is supported, but the entire field must now be interpreted with heightened caution. In light of the retraction, future studies investigating VM in NPC must employ rigorously authenticated cell lines and validate findings across multiple independent models to ensure reproducibility.

Additionally, EBV, via LMP1-activated NF-κB, induces the expression of somatostatin receptor 2 (SSTR2) in NPC. SSTR2 expression is enriched in EBV-positive tumors and serves as a promising diagnostic and therapeutic target, with radiologand uptake on PET-CT imaging and potential for targeted drug conjugates (115–119).

To provide a concise overview of the viral factors implicated in NPC pathogenesis and immune modulation, we summarize the key characteristics, immunomodulatory functions, prognostic associations, and strain variant implications of EBV latent proteins, non−coding RNAs, and emerging lytic−phase gene products in **Table 1**. This table consolidates the evidence discussed in Section 3.2 and serves as a reference for the mechanistic pathways highlighted throughout the review. Given the descriptive nature of many studies on viral polymorphisms, the strength of evidence for each factor varies considerably, and this is noted in the table where applicable.

### 3.3 Engineering the immunosuppressive tumor ecosystem: from cellular dysfunction to five distinct spatial niches

Analysis of 58 studies characterizing the NPC tumor microenvironment (TME) reveals it to be a masterpiece of pathological engineering, actively constructed by the virus-altered tumor cell. Far from a homogeneous “cell soup,” it is a territorially organized, immunosuppressive ecosystem. Based on emerging spatial transcriptomics and multiplex imaging studies, we have synthesized the available evidence into a proposed model comprising five recurrent cellular neighborhoods with distinct functional roles (**Figure 2B**). It should be noted that this model is derived from a limited number of spatial profiling studies and should be considered a preliminary framework that requires further validation in larger, independent cohorts.

The lymphoid compartment within the NPC TME is marked by dysfunction. Advanced single-cell analyses reveal that the CD8+ T-cell compartment exists in a spectrum of dysfunctional states. A significant fraction exhibits a “terminally exhausted” phenotype, defined by high co-expression of multiple inhibitory receptors (PD-1, TIM-3, LAG-3, TIGIT), loss of effector cytokine production, and underlying metabolic defects (56–59). These cells are transcriptionally governed by factors like TOX and NR4A and are largely refractory to reactivation (60). Intertwined with these exhausted effectors is a prominent and active population of regulatory T cells (Tregs). Studies using multi-immunofluorescence accurately define these as CD4+CD25+FOXP3+ cells (65, 66). Their level of infiltration shows a significant positive correlation with plasma EBV DNA load, suggesting that a higher viral burden actively shapes a more immunosuppressive local milieu (63–65). Clinically, higher Treg density is an independent negative prognostic factor and is linked to systemic immune dysregulation (50, 67, 68). Their suppressive mechanisms are multifaceted, including consumption of interleukin-2 (IL-2), direct cytolytic activity, and secretion of immunosuppressive cytokines like IL-10 and TGF-β (69, 70). Recent evidence (71) has further elucidated the molecular pathways linking EBV to Treg activation. The CD70-CD27 axis has emerged as a potential direct mechanism: EBV-encoded LMP1 can upregulate CD70 expression on tumor cells and antigen-presenting cells, which engages CD27 on Tregs, providing costimulatory signals that promote Treg proliferation and enhance their suppressive function (71). While this pathway has been characterized in EBV-associated lymphomas and *in vitro* models, its specific role within the NPC microenvironment remains to be directly investigated and represents an important area for future research. B lymphocytes constitute a heterogeneous group. While a high general abundance of intratumoral B cells is associated with better prognosis, specific subsets, such as IgD−CD27− double-negative B cells, correlate with worsened outcomes (84–87). EBV can directly interact with B cells to suppress immunity; for example, EBV-induced miR-21 can increase B-cell production of IL-10, which in turn suppresses cytotoxic CD8+ T cell activity (88, 89). Natural Killer (NK) cells, though present, often exhibit functionally impaired or “exhausted” states within the NPC TME, characterized by high expression of inhibitory receptors like TIGIT and LAG3, which limits their tumor-killing capacity (61, 62, 90–94).

The myeloid compartment forms a central pillar of the suppressive network. Tumor-associated macrophages (TAMs) are skewed overwhelmingly toward an M2-like, alternatively activated phenotype associated with tissue remodeling and immune suppression (40, 69). Single-cell studies have identified specific subsets, including SPP1+ (osteopontin) TAMs linked to matrix remodeling and C1Q+ TAMs associated with immunosuppressive functions (72). These TAMs execute immune paralysis by expressing enzymes like Arginase-1 (ARG1) that deplete critical nutrients like L-arginine, needed for T-cell function, and by secreting IL-10 and TGF-β (73–75). Myeloid-derived suppressor cells (MDSCs) are another crucial immunosuppressive population. Their expansion is directly linked to the LMP1-induced glycolytic switch in tumor cells, as described earlier, creating a pathogenic LMP1-glycolysis-MDSC axis that correlates with poorer patient survival (31, 76–78).

The stromal compartment, particularly cancer-associated fibroblasts (CAFs), acts as a formidable physical barrier. Activated by cytokines like TGF-β from the viral-inflammatory milieu, CAFs deposit a dense, cross-linked extracellular matrix rich in collagens and fibronectin (70, 79, 80). This forms a “stromal barrier” that physically excludes cytotoxic CD8+ T lymphocytes from the tumor nests, a phenomenon known as immune exclusion (81, 82). Beyond barrier formation, CAFs are active signaling hubs, secreting additional immunosuppressive and pro-angiogenic factors such as CXCL12 and VEGF (83).

The latest paradigm shift lies in understanding the spatial architecture of this ecosystem. Techniques like spatial transcriptomics and multiplexed ion beam imaging are now revealing distinct ‘cellular neighborhoods’ with unique functional roles (4, 137, 138). Molecular subtyping of NPC reveals distinct tumor landscapes between EBV-endemic and non-endemic regions, with significant implications for tailored treatment modalities. Based on synthesis of available evidence from a limited number of spatial profiling studies, we propose a preliminary model comprising five recurrent spatial neighborhoods (**Figure 2B**), which requires further validation in larger cohorts:

1. Immune-Excluded Fibrotic Stromal Neighborhood (at the invasive tumor front): Dominated by CAFs and dense extracellular matrix (collagen, fibronectin), this niche physically traps CD8+ T cells, preventing their infiltration into tumor nests. In this context, “immune exclusion” refers specifically to the barrier-mediated retention of T cells within the stromal compartment, preventing their contact with tumor cells. This pattern has been associated with resistance to immunotherapy in several cancer types (4, 62), though direct evidence in NPC remains limited.
2. Immunosuppressive Interface Neighborhood (at the tumor–stroma junction): Characterized by direct, synapse-like interactions between PD-L1+ tumor cells/TAMs and clusters of exhausted PD-1+ CD8+ T cells. This is the primary site of immune checkpoint engagement and T-cell dysfunction (31, 57, 139).
3. Tertiary Lymphoid Structures (TLS): Organized aggregates of B cells, T cells, and dendritic cells that resemble lymph nodes. In NPC, the presence of TLS has been correlated with improved survival and enhanced response to immune checkpoint inhibitors, suggesting they serve as sites of effective immune priming (6, 84, 108–110). Importantly, the maturation status of TLS appears to be a critical determinant of their functional impact. Mature TLS containing germinal centers and follicular dendritic cells are associated with superior outcomes following ICB, likely due to enhanced local T and B cell priming, whereas immature TLS aggregates lacking organized germinal centers may contribute to immune suppression rather than effective anti-tumor immunity. These findings suggest that not only TLS abundance but also their functional subtypes should be considered as potential predictive biomarkers for immunotherapy response.
4. Vascular Niche Neighborhood: Organized around dysfunctional tumor vessels, often enriched for Tregs and MDSCs that regulate immune cell trafficking and maintain an immunosuppressive perivascular environment (40). The angiogenic factors secreted within this niche, including VEGF, further contribute to immune suppression by inhibiting dendritic cell maturation and promoting PD-L1 expression on endothelial cells.
5. Tumor Core Neighborhood: Densely packed tumor cells with variable immune infiltration, often characterized by hypoxia and metabolic stress that further suppress immune function. The degree of immune cell infiltration within the tumor core can vary substantially; some cores exhibit paucity of T cells (reflecting poor recruitment rather than active exclusion), while others show moderate infiltration with profoundly suppressed effector function due to hypoxia and metabolic constraints. This distinction is important to avoid conflating immune exclusion with immune desert—the former referring to active barrier-mediated retention of T cells in the stroma, the latter to a generalized lack of immune cell recruitment.

This spatial partitioning has direct therapeutic implications. For example, the immune-excluded phenotype may require stroma-disrupting agents (e.g., TGF-β inhibitors, FAP-targeted therapies) before immunotherapy can be effective, while TLS+ tumors with mature, functional structures may be more responsive to immune checkpoint blockade alone (140), though it must be emphasized that the evidence for such spatially-guided therapeutic decisions in NPC is currently preliminary and largely extrapolated from other tumor types.

The composition and functional states of immune cells within the NPC TME are summarized in **Table 2**, which highlights the phenotypic markers, immunosuppressive mechanisms, spatial localization, and potential therapeutic targeting strategies for each major cell type. This table synthesizes the evidence discussed in Section 3.3 and provides a framework for understanding the cellular basis of EBV−driven immunosuppression. The spatial localization information in this table reflects data from a limited number of studies and should be interpreted as indicative rather than definitive.

### 3.4 Translating mechanisms into therapies: a multi-layered strategy

Synthesis of 42 therapeutic studies supports a shift from non-specific cytotoxic regimens to precision strategies that target viral dependencies and the suppressive TME across multiple layers. However, a critical appraisal reveals that while preclinical rationale is strong, clinical validation remains at an early stage for most approaches, with small sample sizes and heterogeneous endpoints limiting generalizability. The following sections summarize the current evidence, with explicit attention to the strength of supporting data.

#### 3.4.1 Immune checkpoint blockade

The rationale for immune checkpoint inhibitors (ICIs) in NPC is exceptionally strong, rooted in the virus-induced presence of a pre-existing, antigen-driven yet profoundly suppressed T-cell infiltrate. Clinically, anti-PD-1 monoclonal antibodies have established a new standard of care for recurrent or metastatic (R/M) NPC. Pembrolizumab monotherapy achieved an objective response rate (ORR) of 26% in PD-L1-positive patients in the KEYNOTE-028 trial, providing the first proof-of-concept (8). Nivolumab monotherapy yielded an ORR of 20.5% in heavily pretreated R/M NPC (9). The most impactful advances come from combination with first-line chemotherapy. The combination of camrelizumab with gemcitabine and cisplatin demonstrated an impressive ORR of 91% in advanced disease (10). Similarly, the landmark phase 3 JUPITER-02 trial for toripalimab plus chemotherapy showed a significant improvement in median progression-free survival (11.7 vs. 8.0 months) and a 40% reduction in the risk of death, leading to its regulatory approval (6, 31). More recently, the phase 3 RATIONALE-309 trial evaluated tislelizumab plus chemotherapy in 263 patients with R/M NPC, demonstrating a significant improvement in progression-free survival compared to chemotherapy alone (median 9.6 vs. 7.4 months; HR 0.50, 95% CI 0.37–0.68), with a manageable safety profile (32). Economic evaluations further demonstrate the cost-effectiveness of this regimen as first-line therapy for R/M NPC, supporting its clinical adoption. This trial adds tislelizumab to the growing list of effective PD-1 inhibitors in NPC and reinforces the role of chemo-immunotherapy as first-line standard.

However, primary and acquired resistance is common, driven by alternative immunosuppressive pathways (LAG-3, TIGIT), physical stromal barriers, and metabolic suppression (31, 40, 61, 62). A critical limitation of current ICI trials is the lack of validated predictive biomarkers beyond PD-L1 expression, which has imperfect correlation with response. Furthermore, most trials have enrolled mixed populations without stratification by TME phenotype or viral strain, potentially obscuring differential treatment effects. Consequently, the frontier involves rationally designed combinations. Synergy with platinum-based chemotherapy extends beyond direct cytotoxicity to include immunogenic cell death (21, 24). Combination with anti-angiogenic agents like apatinib aims to normalize the tumor vasculature and improve T-cell trafficking, with a phase II trial of camrelizumab plus apatinib showing an ORR of 65.2% (96, 141). Beyond PD-1/PD-L1, spatial transcriptomic analyses have identified additional immunosuppressive molecules—such as TIGIT, LAG-3, and TIM-3—that are frequently co-expressed on T cells within the immune-excluded stroma or interface niches. These represent logical targets for next-generation combination therapies, though their clinical efficacy in NPC remains to be tested. Future directions include triple combinations and integrating ICI with agents targeting the myeloid compartment (e.g., anti-CSF-1R) (142, 143). However, it must be emphasized that most combination strategies beyond chemotherapy are supported only by early-phase or preclinical data, and their clinical benefit remains unproven.

#### 3.4.2 EBV-specific adoptive cell therapies

The clonal expression of EBV antigens provides ideal tumor-restricted targets for ACT. First-generation autologous EBV-specific cytotoxic T lymphocytes (CTLs) demonstrated safety and proof-of-concept, with a phase II trial combining them with chemotherapy reporting an ORR of 71.4% (144, 145). The field is now dominated by genetically engineered approaches. Chimeric antigen receptor T cells (CAR-T) targeting LMP1 or LMP2, and T-cell receptor transgenic T cells (TCR-T) targeting the intracellular EBNA1, are in early-phase trials (11, 146, 147). These face hurdles intrinsic to solid tumors: the immunosuppressive TME can inactivate infused cells, and antigen heterogeneity or loss poses relapse risks (11, 57). A critical gap is the lack of data on whether infused cells can penetrate the stromal barrier and function within the hypoxic, nutrient-depleted TME. Next-generation “armored” T cells are being engineered to resist TGF-β signaling or to secrete supportive cytokines like IL-15 to improve persistence (31, 70). However, clinical experience with these advanced products in NPC remains extremely limited, and conclusions about efficacy are premature.

#### 3.4.3 Therapeutic vaccination and lytic induction

This layer aims to enhance pre-existing immunity or alter the antigenic landscape. Therapeutic vaccines (peptide-based, dendritic cell, viral-vectored) seek to boost T-cell responses against EBV latency antigens. A phase I trial of a recombinant MVA vaccine encoding EBNA1 and LMP2 (MVA-EL) was well-tolerated and induced significant antigen-specific T-cell expansion (67, 148, 149). However, durable objective responses have not yet been demonstrated in larger trials. A conceptually distinct strategy is lytic induction therapy, using low-dose epigenetic modulators like decitabine to reactivate the viral lytic cycle. This forces tumor cells to express highly immunogenic lytic antigens. Clinically, the combination of low-dose decitabine with camrelizumab or toripalimab has yielded encouraging response rates (up to ~70% ORR) in Phase II trials for R/M NPC, positioning it as a promising chemo-free regimen (113, 124). However, these are predominantly single-arm, small-sample studies, and the optimal dosing schedule for decitabine—balancing lytic induction with potential immunosuppressive effects—remains to be determined. Therefore, these results should be viewed as preliminary and require confirmation in randomized trials.

#### 3.4.4 Targeting the stromal and metabolic support system

Dismantling the physical and chemical barriers of the TME is crucial for the success of immune-based therapies. Strategies include inhibitors of the colony-stimulating factor 1 receptor (CSF-1R) to deplete or repolarize TAMs, and antibodies against fibroblast activation protein (FAP) or inhibitors of TGF-β signaling to disrupt CAF barriers (40, 70, 131). As noted in Section 3.2, the evidence for vasculogenic mimicry as a therapeutic target requires careful re-evaluation following the retraction of a key study (134). While independent studies support a role for EBV in VM through alternative mechanisms (8, 40, 136), the field lacks consensus on the relative contribution of VM versus classical angiogenesis in NPC progression, and no therapeutic interventions specifically targeting VM have yet entered clinical testing. Preclinical studies targeting VM have shown promise—for example (40), demonstrated that exosome-delivered antagomiRs against miR-BART1-5p inhibited both VM and angiogenesis in NPC models, while (136) revealed the immunomodulatory mechanisms linking EBV-driven VM to immune evasion—but these findings remain at the preclinical stage and have not been validated clinically. Targeting the metabolically hostile TME via inhibitors of the IDO1/TDO or adenosine pathways aims to reverse nutrient deprivation and immunosuppressive metabolite accumulation (93, 150–153). While these agents have shown limited monotherapy efficacy, their true potential lies as sensitizers for ICI or ACT (31, 40), though this remains largely theoretical for NPC.

**Figure 3:**
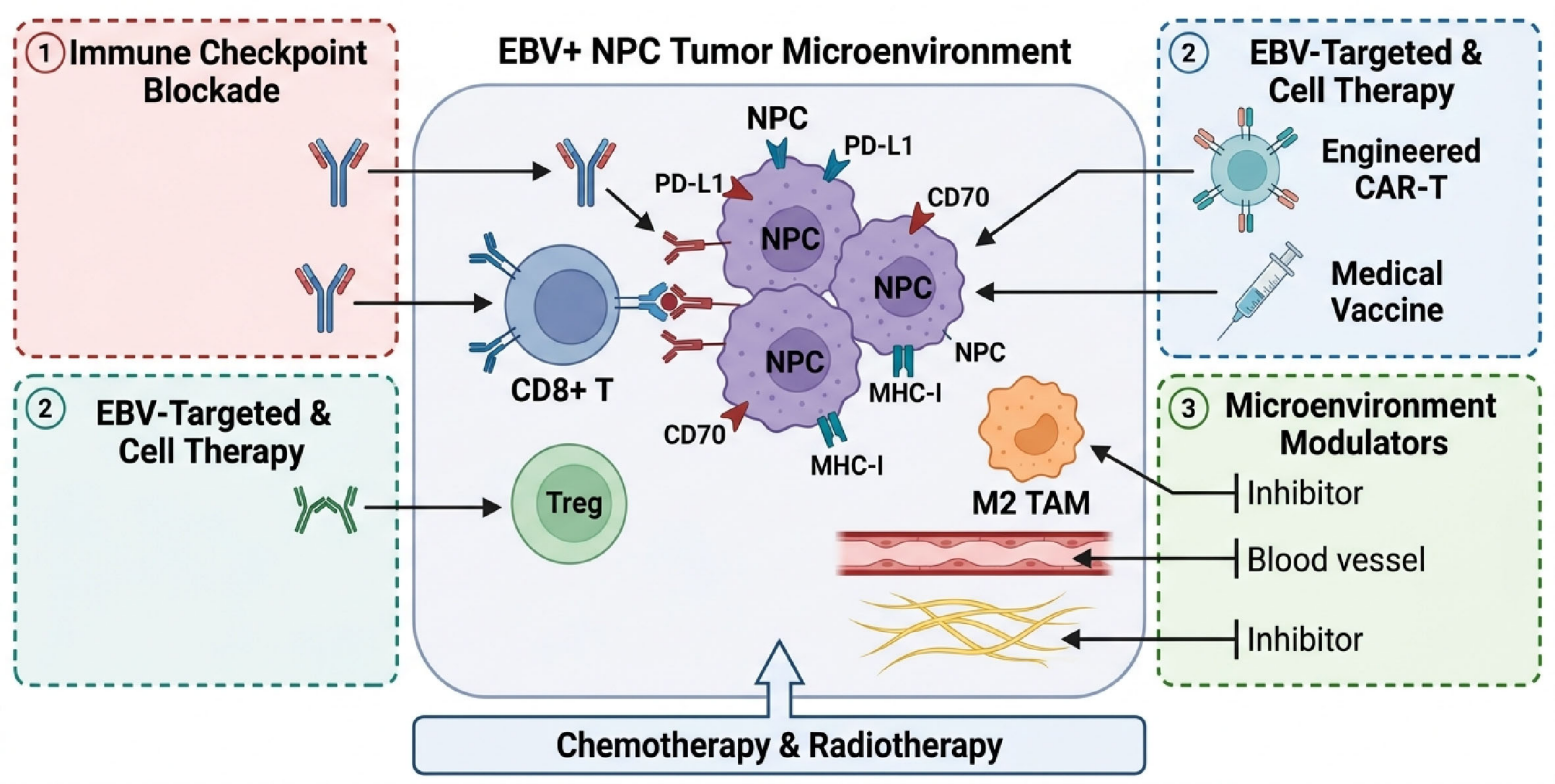
Schematic overview of therapeutic strategies targeting the EBV-driven immunosuppressive tumor microenvironment in NPC. This schematic illustrates the complex interaction between multi-layered therapeutic approaches and the cellular and molecular components of the EBV^+^ nasopharyngeal carcinoma (NPC) tumor microenvironment (TME). The strategies are classified into three key areas, indicated by different colored dashed boxes and numbers: (1) Immune Checkpoint Blockade (red box), using antibodies to target and neutralize specific inhibitory receptors and ligands; (2) EBV-Targeted & Cell Therapy (blue and green boxes), which include antigen-specific approaches such as Engineered CAR-T cells and Medical Vaccines, as well as other antibody-based or T-cell-based therapies; and (3) Microenvironment Modulators (green box), utilizing specific inhibitors to target and counteract immunosuppressive factors, such as those that promote M2 TAM polarization, pathological angiogenesis (Blood vessel formation), and a dense, pro-fibrotic fibrous stroma (Inhibitor pointing to yellow fibrous bands). Arrows indicate the direct targeting and inhibitory effects of each therapeutic modality on specific cell types (purple NPC cells with PD-L1, CD70, and MHC-I surface markers; blue CD8+ T cells; green Treg cells; and orange M2 TAM), as well as on key surface and secreted molecules (e.g., PD-L1, CD70, MHC-I), creating a comprehensive view of how current and emerging therapies are designed to reverse immune evasion and promote tumor eradication in NPC. The bottom block highlights the foundational roles of Chemotherapy & Radiotherapy.

The clinical activity of immune checkpoint inhibitors in recurrent/metastatic NPC is summarized in **Table 3**, while **Figure 3** provides a broader conceptual framework of emerging therapeutic targets that extend beyond PD−1/PD−L1 blockade. Together, these complementary representations illustrate both the established standard of care and the investigational strategies currently under evaluation. It is important to note that the phase 3 results underpinning the standard of care, although robust, are derived from selected patient populations and may not fully generalize to all NPC subtypes or geographic settings. Furthermore, the lack of validated predictive biomarkers beyond PD−L1 expression remains a critical barrier to the personalized application of these therapies.

To provide a synthesized visual overview of the multi-layered therapeutic strategies that target EBV-driven immunosuppression within the NPC tumor microenvironment, we present a schematic diagram (**Figure 3**). This figure maps the key cellular and acellular components of the TME alongside their corresponding targeted interventions, highlighting how distinct spatial niches and molecular pathways can be therapeutically exploited.

#### 3.4.5 Immunomodulatory effects of standard chemoradiotherapy

As anti-PD-1 therapy is typically used as an adjuvant to chemoradiotherapy, understanding how standard treatments modulate the immune microenvironment and EBV biology is essential. Recent translational evidence suggests that gemcitabine and platinum-based chemotherapy can reshape the NPC microenvironment, for instance by modulating myeloid-derived suppressor cell abundance and inflammatory cytokine profiles, thereby potentially creating a more favorable context for subsequent immunotherapy. Furthermore, radiotherapy induces immunogenic cell death, releasing tumor-associated antigens including EBV proteins, and can activate the cGAS-STING pathway, thereby promoting type I interferon responses. However, chemoradiotherapy may also transiently reactivate EBV lytic gene expression, which could either enhance immunogenicity through increased viral antigen presentation or contribute to immune evasion depending on the specific cellular context. The net effect of these immunomodulatory changes on anti-PD-1 response remains an active area of investigation, and the optimal sequencing of chemoradiotherapy with immunotherapy has not been definitively established. This underscores the need for clinical trials specifically designed to evaluate the timing and combination strategies of these modalities.

### 3.5 Biomarkers and the path to precision medicine

Analysis of 35 biomarker studies highlights an evolution from solitary, static biomarkers toward dynamic, multi-modal integration for personalized therapy. However, the majority of these biomarkers lack prospective validation in interventional trials, and their clinical utility remains to be demonstrated. The following sections describe the current landscape, with a clear delineation between established tools and emerging, exploratory markers.

#### 3.5.1 Virological and serological biomarkers

Circulating cell-free EBV DNA remains the most powerful and clinically validated biomarker in NPC. Its utility spans prognostication, response assessment (kinetic clearance is a strong predictor of outcome), and disease surveillance (2, 68, 97). Its fragmentomic profile (size distribution patterns) may provide additional information on therapy-induced cell death mechanisms (1, 98). For screening and early diagnosis, serological antibodies (VCA-IgA, EBNA1-IgA, EA-IgA) are commonly used, with combinations improving sensitivity (99–102). A novel biomarker, the anti-BNLF2b total antibody (P85-Ab), demonstrated higher sensitivity (97.9%) and specificity (98.3%) in a large-scale prospective screening program, outperforming traditional antibody panels (103–106).

Emerging evidence suggests that viral genetic markers may also have prognostic value. Ding et al. identified EBV genome−guided molecular subtypes of NPC based on viral gene expression patterns, which correlated with distinct immune profiles and clinical outcomes (1). Specifically, polymorphisms in lytic−phase genes have been associated with differential immune−related gene expression. BALF2 high−risk strains significantly alter immune pathway expression (54), and population−specific variations in BALF2, BZLF1, and BRLF1 have been observed across diverse ancestries in Southeast Asia (55, 154), suggesting that viral genotyping—including assessment of both latent and lytic gene variants—could hypothetically inform patient stratification. However, it must be reiterated that these genetic associations are purely correlative and lack any functional validation; they are strictly hypothesis−generating and not ready for clinical implementation.

#### 3.5.2 Tissue-based and spatial biomarkers

While PD-L1 expression by Combined Positive Score (CPS) is a standard companion diagnostic, its predictive value in NPC is imperfect, necessitating more sophisticated assays (31, 107). Multiplex immunohistochemistry or imaging mass cytometry allows for the simultaneous quantification of multiple markers, defining not just density but also the spatial relationships of immune cells. Key metrics include the proximity of CD8+ T cells to tumor cells, the classification into “cellular neighborhoods” (e.g., inflamed, excluded, desert), and the presence of tertiary lymphoid structures (TLS), the latter being associated with improved survival and better response to ICI (4, 6, 57, 62). As noted in Section 3.3, the maturation status of TLS (e.g., presence of germinal centers) is likely a more refined predictor than TLS density alone. The density of tumor-infiltrating Tregs (CD4+CD25+FOXP3+), quantifiable via multiplex IHC, correlates with higher EBV DNA load and worse progression-free survival, providing both prognostic and immune monitoring value (63–66). However, the routine clinical application of these spatial metrics is currently limited by the lack of standardized analytical pipelines and prospective validation.

#### 3.5.3 Emerging liquid biopsy and integrative approaches

The exploration of extracellular vesicle (EV)-associated viral proteins is a promising new avenue. The EBV tegument protein BRRF2, actively loaded into EVs by tumor cells, can be detected in patient circulation (111, 155, 156). Higher pre-treatment levels of BRRF2-positive EVs are associated with poorer clinical responses to anti-PD-1 therapy, highlighting its potential as a predictive biomarker of immune evasion and resistance (111, 112). Circulating tumor DNA (ctDNA) analysis via next-generation sequencing can track clonal evolution and detect emerging resistance mutations during therapy (1, 113, 114). To fully exploit these multi-dimensional data, computational frameworks originally developed for other cancers are being explored. For example, TIDE (Tumor Immune Dysfunction and Exclusion) (156) and CIBERSORTx can deconvolve immune cell composition and predict immunotherapy response from bulk or single−cell transcriptomic data. Integrating such tools with EBV−specific signatures (viral gene expression, lytic gene polymorphisms) (155) and spatial features may enable more precise stratification in NPC, though this integrative approach remains at an early research stage and has not been clinically validated. The future lies in integrating these diverse data streams—dynamic virologic, genomic, immunologic, and proteomic—via machine learning to generate predictive models for adaptive therapy selection (1, 113).

A summary of EBV−related biomarkers and their clinical utility is provided in **Table 4**. This table categorizes biomarkers into virological, serological, tissue−based, liquid biopsy, and metabolic imaging modalities, along with their evidence levels, to guide integration into precision oncology frameworks. It should be noted that many of the emerging biomarkers, particularly those based on viral genetic variants or EV−associated proteins, are supported only by single, small−scale studies and should be considered investigational.

**Figure 4:**
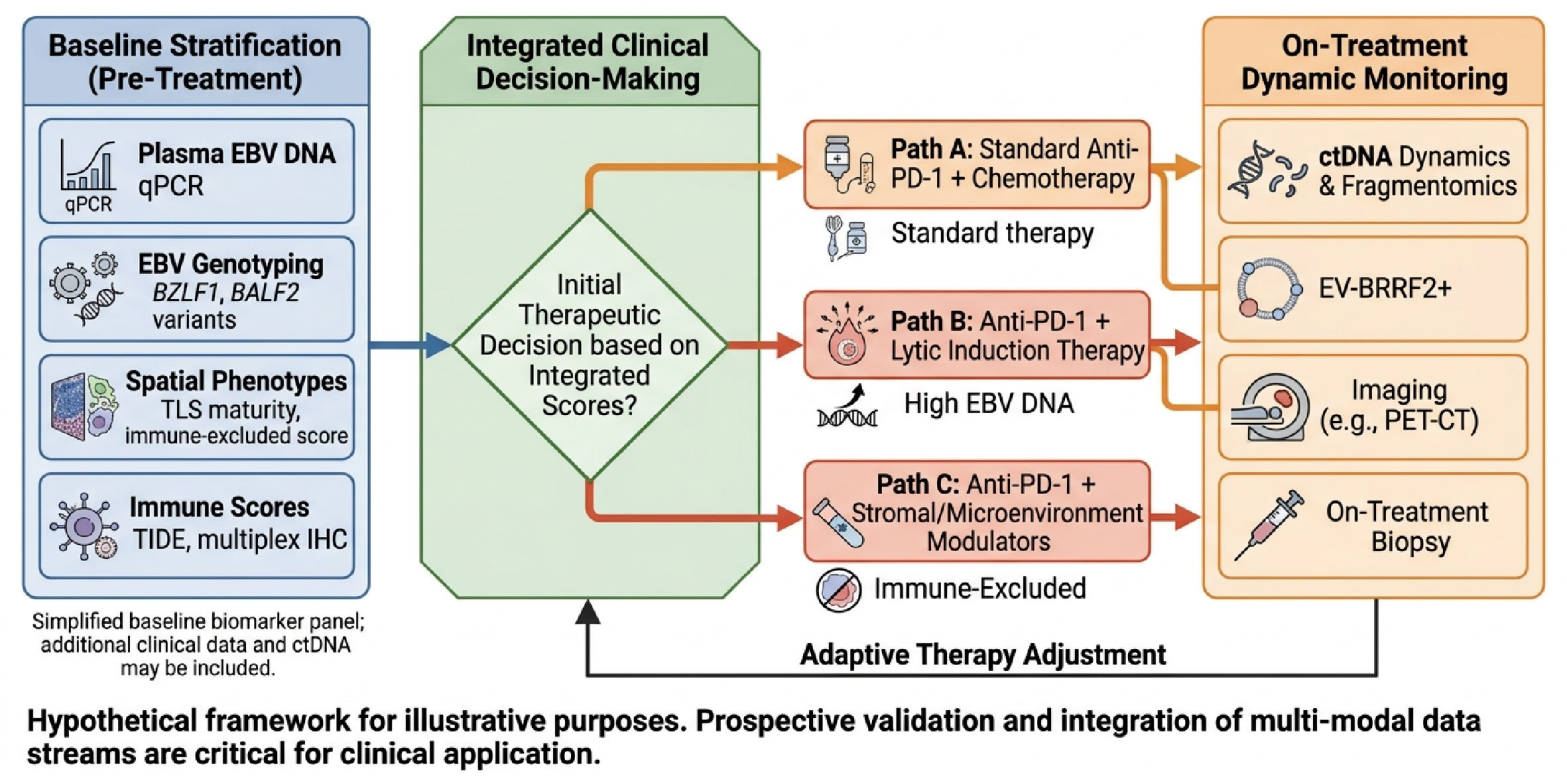
A proposed biomarker-integrated precision oncology framework for EBV-associated NPC. The schematic depicts a future workflow in which baseline biomarkers (EBV DNA, viral genotypes, TME spatial phenotypes, and immune scores) are integrated with on-treatment dynamic monitoring (ctDNA kinetics, EV-BRRF2, imaging) to guide the selection and adaptation of therapeutic strategies. This framework is currently hypothetical and requires prospective validation. Key specific biomarkers and genotypes (e.g., BALF2, BZLF1, BRRF2) are illustrative of the integration approach, and the actual clinical implementation would depend on the results of prospective validation and trial designs.

**Figure 4** summarizes the proposed framework for integrating multi-modal biomarkers to guide precision immunotherapy in EBV-associated NPC. This hypothetical workflow illustrates how pre-treatment and on-treatment biomarkers may be combined to enable adaptive therapeutic decision-making.

## 4 Discussion

This comprehensive review consolidates current evidence from 182 studies, robustly supporting the paradigm that EBV functions as a central, persistent orchestrator—an “immunologic puppeteer”—in NPC pathogenesis. The findings affirm that the virus’s role extends far beyond initiation, involving continuous editing of the host cell’s molecular circuitry and the construction of a complex, spatially organized immunosuppressive tumor ecosystem. By organizing the evidence into a hierarchical framework of viral effectors, downstream signaling, and functional outputs (**Figure 2A**), and by synthesizing spatial data into a proposed preliminary model of five distinct cellular neighborhoods (**Figure 2B**), we provide a comprehensive and conceptually accessible model of EBV-driven TME remodeling.

The strength of evidence is particularly high for the constitutive signaling driven by LMP1 and LMP2A, the immune-evasive functions of BART miRNAs, and the characterization of an exhausted T-cell compartment alongside expanded suppressive myeloid and stromal cells. The clinical success of PD-1/PD-L1 blockade validates the existence of a pre-formed but quenched antiviral immune response, a direct consequence of the viral presence. The addition of the RATIONALE-309 trial to the evidence base confirms that the benefit of chemo-immunotherapy is a class effect among PD-1 inhibitors, further solidifying this approach as standard first-line therapy for R/M NPC.

However, this review also identifies critical limitations in the current evidence base. First, while the immunomodulatory roles of EBV latent proteins are well-established mechanistically, many studies rely on overexpression systems or EBV-negative cell lines reconstituted with individual viral genes, which may not fully recapitulate the complex viral–host dynamics of natural infection. Second, the field has been predominantly descriptive, with an abundance of correlative studies linking viral factors to immune phenotypes but a paucity of functional validation using isogenic viral systems or *in vivo* models that capture TME complexity. Third, clinical trials of emerging therapies—particularly ACT, vaccines, and TME modulators—are predominantly early-phase, single-arm studies with small sample sizes, limiting conclusions about efficacy. Fourth, the recent retraction of a high-profile study on vasculogenic mimicry (134) underscores the importance of rigorous cell line authentication and image integrity checks, and highlights the need for independent validation of key findings across multiple laboratories. The VM field in NPC must now move forward with heightened methodological rigor.

The review identifies several key translational implications. First, combination therapies are not just additive but rational. The most promising strategies logically target different layers of the problem elucidated by the evidence: combining immune checkpoint blockade (to release T-cell brakes) with lytic induction therapy (to enhance antigenicity) or with stromal-modulating agents (to improve T-cell infiltration). Early trial data for combinations like decitabine plus anti-PD-1 support this approach (113, 124). Second, biomarker development must move beyond PD-L1. While plasma EBV DNA remains indispensable for dynamic monitoring, spatial profiling of the TME (e.g., determining immune-inflamed vs. immune-excluded phenotypes) and novel liquid biopsy markers like BRRF2+ EVs offer more nuanced predictors of response and resistance, enabling true personalization (4, 111, 112). Furthermore, emerging data on EBV strain variation—particularly BALF2 polymorphisms—suggest that viral genotyping could theoretically become an additional layer of precision medicine, though this remains highly speculative and requires prospective validation, including functional studies to establish causality (1, 20). Third, next-generation cellular therapies require “armoring.” The detailed mapping of the suppressive TME explains the challenges faced by adoptive T-cell transfer. Engineering EBV-specific CAR-T or TCR-T cells to resist TGF-β, secrete supportive cytokines, or locally degrade stromal barriers is a logical and necessary step informed by this mechanistic understanding (11, 31, 70).

The review also highlights critical gaps and future research priorities. First, the systematic application of spatial multi-omics (e.g., single-cell spatial transcriptomics, high-plex cyclic immunofluorescence) is essential to move from correlative spatial descriptions to causative understanding. Definitive mapping of ligand–receptor interaction networks within specific “cellular neighborhoods” will identify novel, spatially constrained therapeutic targets (4, 62, 157, 158). Second, longitudinal liquid biopsy studies are needed to validate the clinical utility of dynamic biomarkers like EBV DNA fragmentomics or EV-associated proteins for guiding real-time, adaptive therapy decisions, moving from prognostic tools to prescriptive algorithms (1, 98, 159). Third, the development of therapeutic strategies must be increasingly mechanism-informed. This includes creating more potent “armored” cellular therapies, optimizing vaccine platforms and adjuvants for the chronically antigen-exposed setting, and exploring epigenetic drugs that can reverse both viral latency and T-cell exhaustion programs (8, 127, 130, 160–162). Fourth, the role of viral genetic variation in modulating immune responses requires systematic investigation using well-characterized viral isolates and isogenic systems. Studies should move beyond descriptive associations to functional dissection of how specific polymorphisms—not only in BALF2 but also in other lytic−phase regulators such as BZLF1 and BRLF1—impact immune recognition, TME composition, and therapeutic response across diverse populations (27, 50, 54, 55, 154). A comprehensive approach that encompasses both latent and lytic gene variants will be essential to fully capture EBV’s immunomodulatory potential. Fifth, to bridge the gap between descriptive associations and causative mechanisms, future studies should employ humanized mouse models engrafted with patient-derived organoids (PDOs) and autologous immune cells, as well as antigen-specific T cell–tumor spheroid co−cultures. Such systems would permit functional dissection of immune evasion mechanisms in a setting that more closely mimics the native NPC microenvironment. CRISPR−based isogenic EBV mutants will be essential to directly test the functional impact of specific viral polymorphisms on immune phenotypes. Finally, to efficiently test the multitude of rational combinations emerging from this knowledge, innovative adaptive platform trial designs will be crucial. These trials allow for the simultaneous evaluation of multiple biomarker-directed therapy arms against a common control, accelerating the identification of effective regimens for specific molecular and immune subsets of NPC (31, 113, 163–165).

This review has limitations, inherent to its design. As a comprehensive narrative review, it did not include a quantitative meta−analysis, and the synthesis relies on qualitative assessment of heterogeneous studies. The literature search, while systematic in approach, was not prospectively registered. Publication bias may exist, with positive or novel findings more likely to be published (166–168). The field is advancing rapidly, particularly in spatial biology (169, 170) and liquid biopsy (171–173), meaning that evidence is continuously emerging beyond our search cutoff date. A rapid literature checked up to May 2026 did not identify major practice−changing updates. The retraction of reference (134) necessitated a careful re-evaluation of the vasculogenic mimicry evidence; while we have cited alternative studies supporting a role for EBV in VM, the overall strength of this evidence is diminished, and conclusions should be drawn cautiously. Additionally, many of the mechanistic models of TME organization (173–179) (e.g., the five−neighborhood framework (175, 180)) are based on a limited number of studies (181–183) and should be regarded as provisional until validated by larger (184), multi−center spatial profiling efforts (185–188).

In conclusion, this comprehensive review synthesizes robust evidence that EBV is a master regulator of NPC, sculpting both the tumor cell and its surrounding immunosuppressive landscape. By critically appraising the evidence, identifying key gaps, and proposing a hierarchical and spatial framework for understanding viral–host interactions, we provide a roadmap for future research. The detailed elucidation of these mechanisms reveals a spectrum of therapeutic vulnerabilities. The path forward lies in leveraging this knowledge to develop and test precision oncology strategies that combine immune activation, viral targeting (including consideration of viral strain) (189), and microenvironment modulation, guided by sophisticated, integrated biomarker profiling. By embracing spatial biology, dynamic monitoring, rigorous functional validation, and innovative trial designs, the management of EBV-associated NPC can transition from a generalized approach to a dynamically personalized, mechanism-driven paradigm aimed at definitively countering this viral architect of cancer.

## Supplementary material

Supplementary Table 1 Detailed search strategy for PubMed/MEDLINE.

Supplementary Table 2 Summary of risk of bias assessment.
