# Supplementary material for: Epstein–Barr virus-driven immunosuppression in nasopharyngeal carcinoma: a comprehensive review of viral mechanisms, spatial tumor ecosystems, and precision therapeutics
Source: Front Immunol. 2026 Jun 17;17:1875687. doi: 10.3389/fimmu.2026.1875687 (PMC13318586; doi:10.3389/fimmu.2026.1875687)
Supplement: Supplementary Table 1 — Detailed search strategy for PubMed/MEDLINE. [file Table1.docx]

| **Supplementary Table S1. Detailed search strategy for PubMed/MEDLINE** | | |
| --- | --- | --- |
| Search step | Search terms | Results |
| #1 | "Nasopharyngeal Carcinoma"[MeSH] OR "Nasopharyngeal Neoplasms"[MeSH] OR nasopharyngeal carcinoma*[tiab] OR nasopharyngeal cancer*[tiab] OR nasopharyngeal neoplasm*[tiab] OR NPC[tiab] | 28,547 |
| #2 | "Herpesvirus 4, Human"[MeSH] OR "Epstein-Barr Virus"[tiab] OR EBV[tiab] OR "Human Herpesvirus 4"[tiab] OR HHV-4[tiab] | 41,236 |
| #3 | "Tumor Microenvironment"[MeSH] OR tumor microenvironment[tiab] OR tumour microenvironment[tiab] OR TME[tiab] OR immune microenvironment[tiab] OR immunosuppression[tiab] OR immune evasion[tiab] | 189,421 |
| #4 | "Immunotherapy"[MeSH] OR immunotherapy[tiab] OR immune checkpoint inhibitor*[tiab] OR PD-L1[tiab] OR PD-1[tiab] OR adoptive cell therap*[tiab] OR therapeutic vaccin*[tiab] OR lytic induction[tiab] | 172,663 |
| #5 | "Biomarkers, Tumor"[MeSH] OR biomarker*[tiab] OR EBV DNA[tiab] OR circulating tumor DNA[tiab] OR liquid biopsy[tiab] OR spatial transcriptomic*[tiab] | 198,352 |
| #6 | "Signal Transduction"[MeSH] OR NF-kappa B[tiab] OR NF-κB[tiab] OR PI3K[tiab] OR AKT[tiab] OR mTOR[tiab] OR JAK[tiab] OR STAT[tiab] OR LMP1[tiab] OR LMP2[tiab] OR EBNA1[tiab] OR BART[tiab] OR EBER[tiab] OR BALF2[tiab] OR BZLF1[tiab] OR BRLF1[tiab] | 425,678 |
| #7 | #1 AND #2 AND (#3 OR #4 OR #5 OR #6) | 2,186 |
| #8 | #7 AND (english[Filter]) | 1,425 |
| Note: The same conceptual search structure was adapted for Embase (using Emtree terms and equivalent free-text keywords, yielding 1,780 records) and Web of Science Core Collection (yielding 1,030 records), with language filter applied. The total number of records identified from the three databases before deduplication was 4,235. No Cochrane Library search was performed in the original search. All searches were conducted from database inception through December 31, 2025. | | |
