# Supplementary material for: Epstein–Barr virus-driven immunosuppression in nasopharyngeal carcinoma: a comprehensive review of viral mechanisms, spatial tumor ecosystems, and precision therapeutics
Source: Front Immunol. 2026 Jun 17;17:1875687. doi: 10.3389/fimmu.2026.1875687 (PMC13318586; doi:10.3389/fimmu.2026.1875687)
Supplement: Supplementary Table 2 — Summary of risk of bias assessment. [file Table2.docx]

| Supplementary Table S2. Summary of risk of bias assessment | | | | |
| --- | --- | --- | --- | --- |
| Study design | Number of studies assessed | Assessment tool | Key domains evaluated | Overall risk-of-bias judgment |
| Randomized controlled trials (e.g., JUPITER-02, RATIONALE-309) | 5 | RoB 2 | Randomization process, deviations from intended interventions, missing outcome data, measurement of outcome, selection of reported result | Low risk of bias for primary endpoints; some concerns for open-label designs and subgroup analyses |
| Prospective/retrospective cohort studies (e.g., biomarker correlation studies) | 47 | NOS | Selection of exposed/non-exposed, comparability of cohorts, ascertainment of exposure/outcome, adequacy of follow-up | Moderate risk of bias; many studies lacked adjustment for key confounders (e.g., tumor stage, treatment history) and had short follow-up |
| Preclinical in vitro studies | 98 | Customized checklist | Blinding during outcome assessment, independent replication, appropriateness of controls (e.g., isogenic EBV-negative lines, viral strain validation), cell line authentication | Moderate to high risk of bias; the majority did not report blinding, replication was inconsistent, and cell line authentication was rarely documented. Studies using overexpression systems were considered to have limited generalizability to natural infection |
| Preclinical in vivo studies (xenograft/animal models) | 32 | Customized checklist | Randomization to treatment groups, blinding, sample size justification, relevance of animal model to human NPC | High risk of bias; most studies used immunocompromised xenograft models that do not recapitulate the human TME, and few reported power calculations or blinding |
| This table provides a qualitative summary of the risk-of-bias appraisal for the included studies, stratified by study design. The assessment was performed independently by two reviewers using standardized tools: the Cochrane Risk of Bias 2 (RoB 2) tool for randomized trials, the Newcastle-Ottawa Scale (NOS) for observational studies, and a customized checklist for preclinical laboratory studies. Discrepancies were resolved by discussion.  Overall, the body of evidence informing this review is heterogeneous and largely derived from studies with moderate to high risk of bias. Conclusions regarding therapeutic efficacy are primarily supported by a small number of well-conducted randomized trials, while mechanistic and biomarker findings are predominantly based on exploratory, correlative studies that require prospective validation. The risk-of-bias profile of individual studies was considered during the narrative synthesis, with findings from higher-risk studies interpreted more cautiously. | | | | |
